# First Ever Storage of Ultracold Neutrons in a Magnetic Trap Made of Permanent Magnets

**DOI:** 10.6028/jres.110.051

**Published:** 2005-08-01

**Authors:** V. F. Ezhov, A. Z. Andreev, A. A. Glushkov, A. G. Glushkov, M. N. Groshev, V. A. Knyazkov, G. B. Krygin, V. L. Ryabov, A. P. Serebrov, B. A. Bazarov, P. Geltenbort, F. J. Hartman, S. Paul, R. Picker, O. Zimmer, N. A. Kovrizhnykh

**Affiliations:** Petersburg Nuclear Physics Institute, Gatchina, Russia; Research Center “Domen”, S-Petersburg, Russia; Institut Laue-Langevin, Grenoble, France; Technical University, Munich, Germany; Research Institute of Electrophysical Apparatus, S-Petersburg, Russia

**Keywords:** magnetic traps, permanent magnets, ultracold neutrons

## Abstract

Further improvement in the accuracy of any neutron lifetime experiment by means of ultracold neutrons (UCN) in material bottles is limited due to unavoidable systematic effects when the UCN are reflected from the walls. However, such effects can be excluded in principle if magnetic trapping of UCN is used. The storage of UCN in a small magnetic trap made of permanent magnets was demonstrated for the first time ever. The measured storage time in this feasibility study was (882 ± 16) s. At this level of accuracy no depolarization was observed.

## 1. Introduction

The precise measurement of the mean lifetime *τ*_n_ of the free neutron is a low-energy experiment searching for new physics beyond the Standard Model. In recent years the accuracy of *τ*_n_ experiments has been considerably improved by the use of ultracold neutrons (UCN) stored in traps. At the moment the value adopted by the Particle Data Group is *τ*_n_ = (885.7 ± 0.8) s [1]. Limits are imposed, however, by the losses suffered during reflections from the material walls. This systematic problem may be avoided by the use of magnetic traps where wall collisions of the neutrons are prevented.

The first ideas for the magnetic storage of neutrons came from W. Paul and V. V. Vladimirski [2]. It should be pointed out that magnetic trapping is now successfully used in the physics of cold atoms [3]. In a magnetic trap the magnetic field increases in all directions from its center. Neutrons with their magnetic moment directed along the magnetic field lines are subject to a force parallel to the direction of increasing magnetic field and vice versa. A magnetic barrier of 1 T completely reflects the neutrons with velocities below 3.4 m/s. The standard magnetic trap of the Ioffe-Pritchard type that is widely used in atomic physics consists of a magnetic quadrupole with two solenoids at its edges. The quadruple reflects neutrons moving radially and the solenoids those moving in the axial direction.

The first real magnetic trap for neutrons was tested in the eighties [4]. This trap used superconducting magnets. It was not possible at that time and even nowadays is not easy to change the current in the magnetic entrance shutter of such superconducting systems with a speed needed for the lifetime measurements. Hence a complicated experimental setup was used to produce UCN inside the trap [5], using inelastic scattering of neutrons in superfluid He. On the other hand modern technology permits to manufacture traps from permanent magnets with not much smaller values of the magnetic flux density *B* and one may use a normal solenoid as a magnetic shutter. The main aim of this work is to study magnetic UCN trapping systematically and to start measuring the neutron lifetime in the permanent-magnet trap.

The proposed magneto-gravitational trap is a vertical cylinder with a conical lower part [6]. In the cylindrical part of the trap the magnets are magnetized in the horizontal direction and form a twenty-pole magnetic system. A convergent sequel of twenty-pole systems constitutes the conical part. The magnetic flux density at the magnet surface equals about 1 T. An orifice for a neutron guide in the lower conical part of the trap allows one to fill the trap with neutrons and empty it again. A solenoid is used as a magnetic shutter for this neutron guide.

**Fig. 1:**
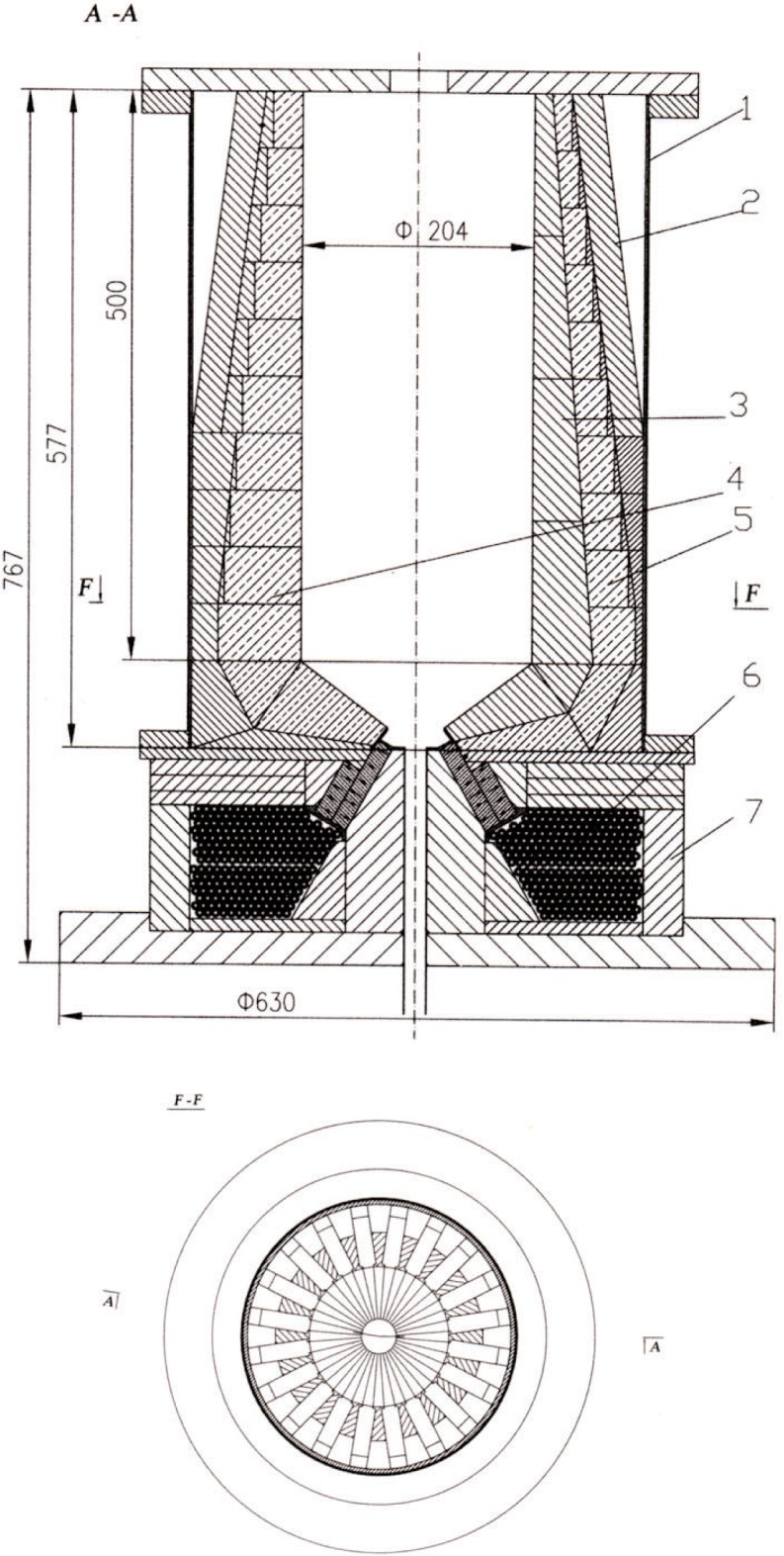
Cross-section of the Magnetic Trap. 1 – Vacuum Cover, 2 – Yoke of Permanent Magnets, 3 – Poles, 4 – Main Magnets, 5 – Additional Magnets, 6 – Solenoid, 7 – Yoke of Solenoid.

The cross-section of the magnetic trap is shown in Fig. 1. The main part consists of 560 small permanent magnets with horizontal magnetization and FeCo poles between them. Neighboring magnets are magnetized in opposite directions. The main parameters of these magnets are *B*_r_ ≥ 1.2 T and *H*_cm_ ≥ 1800 kA/m. Such large values of *B*_r_ and *H*_cm_ permit one to obtain a magnetic flux density near the wall of about 1 T and to create a field gradient of about 2 T/cm.

The experimental scheme is the same as that for material traps. After filling the trap with UCN one waits some time in order to clean the neutron spectrum from its high-energy components. Afterwards one has to determine the number of trapped neutrons as a function of their storage time. Previous experiments with material traps showed that the main systematic effects could be eliminated if one had the possibility to compare analogous results for UCN with different energy spectra. All of these ideas are implemented into the experimental scheme proposed here.

**Fig. 2:**
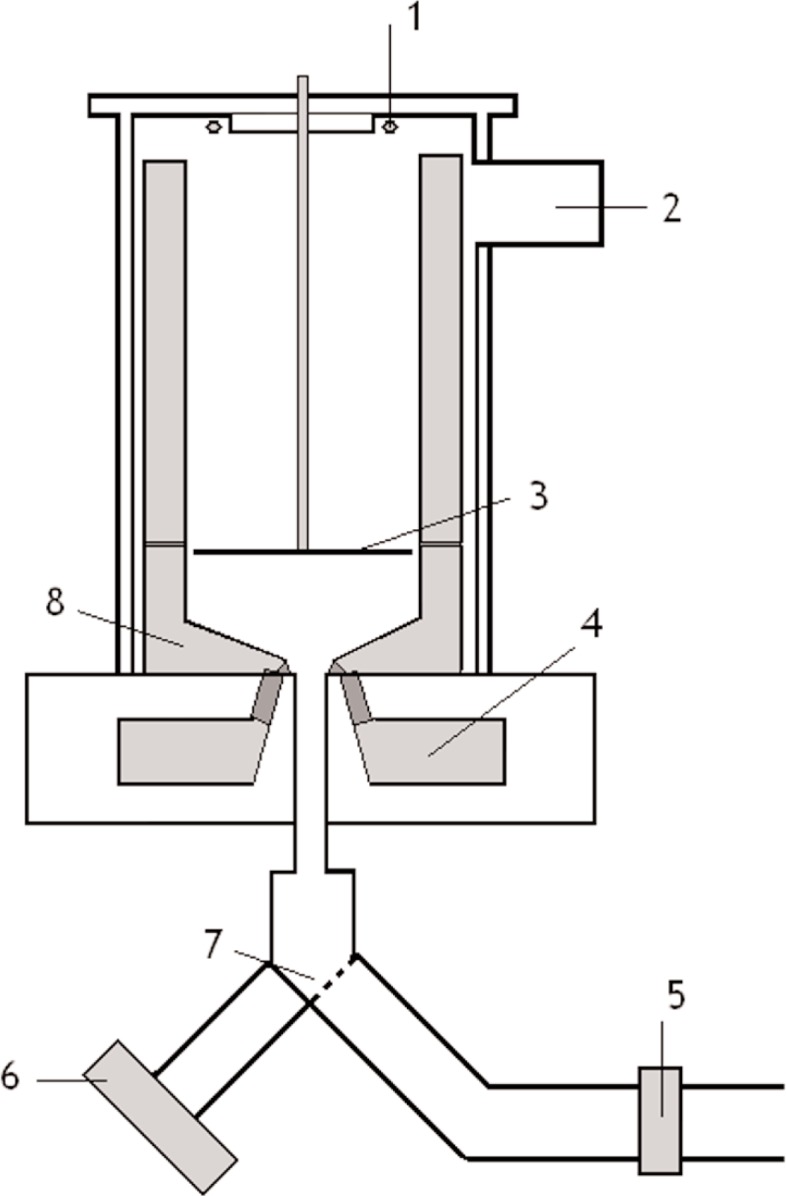
Scheme of Experimental design. 1 – Additional Coil, 2 – Vacuum Pumping, 3 – Neutron Absorber, 4 – Solenoid, 5 – Neutron shutter, 6 – Detector, 7 – Neutron Switcher, 8 – Magnets and Poles.

The most important features of our design (Fig. 2) are the following:

1. The trap walls consist of a periodic structure with a characteristic period of ~1 cm. The magnetic field decreases quite fast (gradient ≈2 T/cm). Due to the concentration of the field in a small volume, the required magnetic material is minimized and the effective trap volume may be increased.
2. The UCN are transferred to the trap through a neutron guide inside the solenoid at the bottom. After loading the trap this entrance is closed by switching on the current in the solenoid. To facilitate fast operation, we use a normal-conducting solenoid with iron core and permanent magnets.
3. UCN with energies exceeding the solenoid magnetic barrier will penetrate the barrier and disappear. Thus changing the current in the solenoid easily modifies the spectrum of trapped UCN.
4. Moreover, by applying a magnetic barrier at the entrance during trap loading, the spectrum may also be cut from the low-energy side. This flexibility in the choice of the UCN spectrum is very useful for eliminating systematic errors in the neutron lifetime measurement.
5. To avoid UCN depolarization at the points of zero magnetic fields we use the field generated by the lower solenoid, which is orthogonal to the magnetic field from the permanent magnets. For this purpose an iron yoke guides the magnetic field from the solenoid to the top of the trap.
6. To investigate the depolarization of UCN we intend to cover the inner trap walls with Fomblin oil to reflect depolarized UCN. In this case the depolarized UCN penetrate the magnetic barrier inside the solenoid and are measured by the UCN detector installed below the solenoid. Hence this detector may be used as monitor for depolarization losses during neutron storage.

As we mentioned above, neutron losses in the magnetic trap take place predominantly due to their depolarization (i.e., a spin flip relative to the direction of the magnetic field). Let us try to estimate the probability of neutron depolarization in a region with a strong magnetic field. The precession of the magnetic moment is described by

$$\frac{d\vec{\mu }}{dt}=\gamma_{n}\vec{\mu }\times \vec{B}$$ (1)

with *γ*_n_ = 1.83 · 10^8^ s^−1^ T^−1^. The neutron magnetic moment will follow the magnetic field direction if the *adiabatic* condition is fulfilled:

γnB>>dBdtB=ν⋅∇|B|B, (2)

where *ν* is the neutron velocity. Thus for *B* = 1 T, ∇*B* = 1 T/mm and *ν* = 3.4 m/s, one obtains that the adiabatic condition is well fulfilled, 1.83 · 10^8^ ≫ 3.4 · 10^3^. Obviously this condition becomes invalid only in weak magnetic fields (about 10^−4^ T).

To estimate the probability of depolarization in the weak magnetic field we will follow [3]. Let us assume that one component of the magnetic field, for example *H_z_* = const, and another one—*H_x_* will change its direction. In [3] it was shown that the probability of depolarization during one pass near such a point is equal to:

$$w=e^{-\pi \varpi \tau }$$ (3)

where *ω* = *μH_z_/ħ* is the precession frequency of the neutron magnetic moment in *Hz*, and τ= *H_z_/Ḣ*·is an effective rotation time for the magnetic field. If the neutron passes such points of fast magnetic field rotation *N* times, one has to have *N_w_* ≪ 1 in order to fulfill the adiabatic condition. Thus one obtains for the minimum value of the magnetic field

$$\frac{\pi \mu H_{z\;\min}^{2}}{h\dot{H}/2\pi }>\ln N.$$ (4)

Therefore for each value of the effective time of magnetic field rotation one may choose such a value of *H_z_* that depolarization may be neglected. In our trap the lower solenoid and an additional small solenoid at the upper flange create a component *H_z_* that is orthogonal to the magnetic field from the permanent magnets. If the characteristic dimension of field rotation is equal to 3 mm, then the minimum effective time is equal to 10^−3^ s. Hence it is enough to create *H_z_* = 796 A/m (10 Oe) to fulfill the adiabatic condition for 10^5^ passes near a rotating field of ~ 80 A/m (1 Oe). This corresponds to a depolarization probability of ~ 10^−4^/s for the characteristic dimensions of our trap.

In order to check the proposed ideas the more complicated lower part of the trap and the magnetic shutter were manufactured. The height of this part of the trap is 16 cm. The magnetic field created by the shutter in the lowest point of the trap (at a height of 10 mm from the edge of the neutron guide) is equal to 1.4 T. The diameter of the neutron guide was equal to 20 mm.

The operation of this small trap was investigated at the UCN beam of the Institut Laue-Langevin (ILL). To cut the spectrum of stored neutrons a polyethylene absorber was placed inside the trap at a height of 15 cm above its bottom. This resulted in an effective volume of the trap of 3.6 L. The trap was filled for 170 s. The density of stored neutrons reached 0.017 n/cm^3^. The measured storage time in the trap was equal to (882 ± 16) s. The statistical error quoted was obtained in 245 runs lasting 143 h. By searching for an increase of the background counts in the detector during storage we were able to detect depolarized neutrons. At this level of accuracy no depolarization was observed.

To estimate the number of UCN that may be stored inside a trap of 55 cm height, an aluminum cylinder with the diameter of the trap simulated the missing upper part. It was found that the density of neutrons in this extended set-up was equal to 0.11 n/cm^3^. It isn’t a record value of neutron density and it can be increased by more careful assembling of the neutron guide.

Our experiment showed that magnetic trapping is a viable method for neutron lifetime measurements. Now we have increased the height of the trap to 55 cm.

As a next stage we are planning to increase the diameter of the trap by a factor of two. This possibility is based on the similarity relation for magnetic systems. To use the existing magnets and poles in a new setup, one must change the quantity of poles of the trap from twenty to forty. The diameter of the neutron guide will be increased as well. So in this case we’ll repeat the manufacture of a second set of magnets and poles and after that we’ll construct a trap of larger volume from these two sets (new and previous one). The increase in the trap volume will permit us to improve the experimental sensitivity in the storage time measurement to ±1.6 s in a 6 d run. Estimated data for such a trap is shown in the last column of table 1. The possible increase of the UCN density due to a more careful assembly of the neutron guide is not included in this estimation.

The main cost of the trap consists of the cost of the permanent magnets. The main parameters are shown in table 1. It is necessary to point out that the estimations of accuracy are made from experimental data at the UCN beam PF2 at the ILL and there are no corrections made do to the possible increase of UCN density due to the quality of neutron guide or use of another UCN source.

## Figures and Tables

**Table 1:** 

	Volume	Neutrons after 50 s of cleaning time	Neutron density after 50 s of cleaning time (n/cm^3^)	Accuracy of lifetime measuring
Existing lower part of trap	3.61	62.6 ± 2.0	0.017	16 s in 6 days
Upper part of trap	15.61	1770 ± 11	0.11	3.1 s in 6 days
Trap of larger diameter	62.41	7000	0.11	1.6 s in 6 days
